# Rate of Pediatric Appendiceal Perforation at a Children’s Hospital During the COVID-19 Pandemic Compared With the Previous Year

**DOI:** 10.1001/jamanetworkopen.2020.27948

**Published:** 2020-12-04

**Authors:** Rick Place, Jonathan Lee, John Howell

**Affiliations:** 1 Department of Emergency Medicine, Inova Fairfax Medical Campus, Virginia Commonwealth University School of Medicine, Falls Church; 2 Department of Pediatrics, Inova Fairfax Medical Campus, Virginia Commonwealth University School of Medicine, Falls Church

## Abstract

This cross-sectional study assesses the rate of appendiceal perforations during the COVID-19 pandemic at a children’s hospital during10-week periods in 2020 vs 2019.

## Introduction

Clinicians have witnessed a dramatic shift in health care consumption during the coronavirus disease 2019 (COVID-19) pandemic as patients fear exposure to coronavirus from visiting health care facilities.^1,2^ Among the many concerning patterns emerging is that of delayed medical care. Garcia et al^3^ reported a 38% decline in cardiac ST-segment elevation myocardial infarction activations, and Teo et al^4^ noted fewer patients with ischemic stroke presenting within the therapeutic window. Lazzerini et al^5^ described 12 children who presented in severe condition because of delays in accessing care; 4 of these children died.

We noted an increased incidence of perforated appendicitis coinciding with the closure of Virginia public schools on March 16, 2020, and sought to determine whether a true difference existed.

## Methods

As part of a quality improvement initiative to alert our community to the dangers of delayed medical care, we assessed the percentage of acute and perforated appendicitis in children younger than 18 years. The study period covered 10 weeks between March 16 and June 7, 2020, at the pediatric emergency department in Inova Children’s Hospital in Northern Virginia. We compared this rate with the same period 1 year earlier. The final diagnosis of appendicitis and the presence of perforation was determined by the operative report. Data were collected using the electronic medical record (Epic; Epic Systems Corporation). We used the Strengthening the Reporting of Observational Studies in Epidemiology (STROBE) reporting guideline for cross-sectional studies. The office of research at INOVA Health System determined this review to be exempt from the need for study approval or patient informed consent because it was conducted as an institutional quality improvement project.

Nominal outcomes were analyzed using either Fisher exact or χ^2^ tests. Continuous outcomes were analyzed using a 2-tailed unpaired *t* test. The α level was set at 0.05 for all comparisons. Data were analyzed using online GraphPad, version 7.0 software (GraphPad Software).

## Results

During the 10-week study period, 90 children were diagnosed with acute appendicitis; perforation had occurred in 35 cases (39%). The median patient age was 10 years (interquartile range [IQR], 7-13 years), 46 (51%) were boys, and 30 (33%) were White individuals (Table). During the same period in 2019, 70 children presented with acute appendicitis; perforation had occurred in 13 cases (19%). The median patient age in 2019 was 11 years (IQR, 9-14 years), 44 (63%) were boys, and 33 (47%) were White individuals. This change in the number of cases between 2020 and 2019 represents a 20% absolute increase in the incidence of perforated appendicitis (*P* = .009).

**Table.** Demographic Characteristics and Outcomes of Patients With Appendicitis in a 10-Week Period, 2019 vs 2020

Characteristic	No. (%)	
	2019 Control period (n = 70)	2020 COVID-19 period (n = 90)
Males	44 (63)	46 (51)
Age, median (IQR), y	11 (9-14)	10 (7-13)
Race/ethnicity		
White	33 (47)	30 (33)
Black	4 (6)	1 (1)
Latino or Hispanic	8 (11)	13 (14)
Other or unavailable^a^	25 (36)	46 (51)
Perforated appendicitis	13 (19)	35 (39)
Medical management	0	8 (9)

Abbreviations: COVID-19, coronavirus disease 2019; IQR, interquartile range.

^a^ Indicates that the patient selected the “other” category or nothing was selected on registration.

During the COVID-19 study period, 8 children (9%) presented with a pelvic abscess that required initial medical management before delayed interval appendectomy. No patient required medical management in the 2019 control period (Table).

Patient volumes in the emergency department were also decreased during the pandemic, from a mean of 144 patients per day (95% CI, 136%-152%) to 65 patients per day, reflecting a 55% decrease (95% CI, 39%-90%); *P* < .001). However, there was a nonsignificant increase in the admission rate of 16.4% over the 10-week study period compared with a baseline admission rate of 10.5% in 2019 (*P* = .07).

## Discussion

Although studies in the adult literature and case series in the pediatric literature have reported delays in medical care attributable to COVID-19, we report a statistically significant increased rate of appendiceal perforation during this pandemic.^2,3,4,5,6^ Over the 3 months studied, parents displayed visible signs of anxiety when in the emergency department and openly expressed reluctance to visit the hospital for fear of contracting COVID-19.

Unlike their adult counterparts, pediatric patients do not commonly experience medical conditions with a high risk of mortality. However, medical conditions do exist for which delayed diagnosis and management can lead to a significant increase in morbidity, prolonged hospitalization, and increased financial expense. In this cross-sectional study, appendiceal perforation also resulted in pelvic abscess, bowel obstruction, and sepsis.

This was a single institutional cohort, and generalizability to other settings may be limited. Although the reduction in “unnecessary” emergency care may be welcomed by some, broad avoidance of the emergency department may lead to increased morbidity and mortality in both children and adults.
